# 7′-Amino-1′*H*-spiro­[cyclo­heptane-1,2′-pyrimido[4,5-*d*]pyrimidin]-4′(3′*H*)-one

**DOI:** 10.1107/S1600536812031492

**Published:** 2012-07-25

**Authors:** Shu Chen, Daxin Shi, Mingxing Liu, Jiarong Li

**Affiliations:** a School of Chemical Engineering and Environment, Beijing Institute of Technology, Beijing 100081, People’s Republic of China

## Abstract

The title compound, C_12_H_17_N_5_O, was obtained by cyclo­condensation of 2,4-diamino­pyrimidine-5-carbonitrile with cyclo­hepta­none. The tetra­hydro­pyrimidine ring has a dis­torted boat conformation and the cyclo­heptane ring adopts a chair conformation. In the crystal, molecules are linked *via* N—H⋯O and N—H⋯N hydrogen bonds generating a three-dimensional network.

## Related literature

For medicinal and biological properties of 2,3-dihydro­pyrimido[4,5-*d*]pyrimidin-4(1*H*)-one derivatives, see: Gebauer *et al.* (2003 ▶); McDermott *et al.* (2006 ▶). For a related structure, see: Shi *et al.* (2010 ▶).
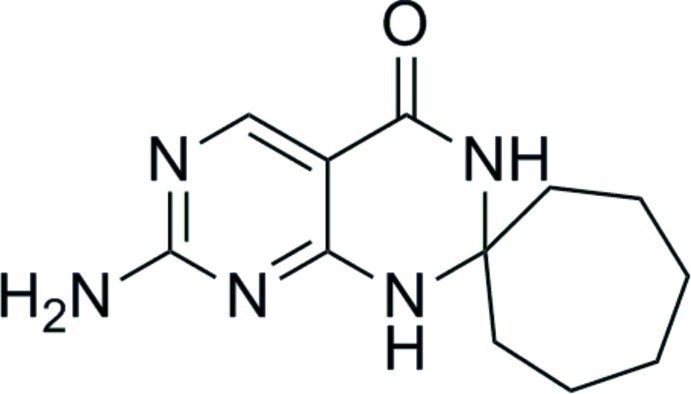


## Experimental

### 

#### Crystal data

C_12_H_17_N_5_O
*M* *_r_* = 247.31
Monoclinic, 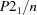

*a* = 10.798 (3) Å
*b* = 10.365 (3) Å
*c* = 11.341 (3) Å
β = 110.287 (4)°
*V* = 1190.5 (6) Å^3^
*Z* = 4
Mo *K*α radiation
μ = 0.09 mm^−1^
*T* = 153 K
0.39 × 0.35 × 0.26 mm

#### Data collection

Rigaku AFC10/Saturn724+ diffractometer
9163 measured reflections
3450 independent reflections
3237 reflections with *I* > 2σ(*I*)
*R*_int_ = 0.036

#### Refinement

*R*[*F* ^2^ > 2σ(*F* ^2^)] = 0.054
*wR*(*F* ^2^) = 0.129
*S* = 1.00
3450 reflections
180 parameters
H atoms treated by a mixture of independent and constrained refinement
Δρ_max_ = 0.35 e Å^−3^
Δρ_min_ = −0.22 e Å^−3^

### 

Data collection: *CrystalClear* (Rigaku/MSC, 2009 ▶); cell refinement: *CrystalClear*; data reduction: *CrystalClear*; program(s) used to solve structure: *SHELXS97* (Sheldrick, 2008 ▶); program(s) used to refine structure: *SHELXL97* (Sheldrick, 2008 ▶); molecular graphics: *CrystalStructure* (Rigaku/MSC, 2009 ▶); software used to prepare material for publication: *CrystalStructure*.

## Supplementary Material

Crystal structure: contains datablock(s) I, global. DOI: 10.1107/S1600536812031492/cv5317sup1.cif

Structure factors: contains datablock(s) I. DOI: 10.1107/S1600536812031492/cv5317Isup2.hkl

Supplementary material file. DOI: 10.1107/S1600536812031492/cv5317Isup3.cml

Additional supplementary materials:  crystallographic information; 3D view; checkCIF report

## Figures and Tables

**Table 1:** Hydrogen-bond geometry (Å, °)

*D*—H⋯*A*	*D*—H	H⋯*A*	*D*⋯*A*	*D*—H⋯*A*
N1—H1N⋯O1^i^	0.88 (2)	2.05 (2)	2.9176 (16)	167 (2)
N2—H2N⋯O1^ii^	0.87 (2)	2.30 (2)	3.1587 (16)	168.3 (17)
N5—H0*B*⋯O1^iii^	0.84 (2)	2.22 (2)	2.9234 (17)	141.4 (19)
N5—H0*A*⋯N3^iv^	0.87 (2)	2.11 (2)	2.9826 (18)	172.5 (17)

Symmetry codes: (i) 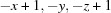; (ii) 
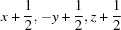
; (iii) 
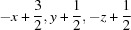
; (iv) 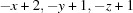.

**Fig. 1.**
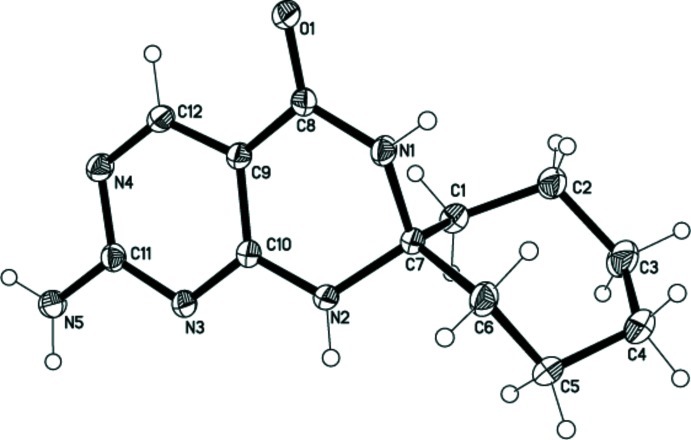
The molecular structure of the title compound with the atom numbering scheme. Displacement ellipsoids are drawn at the 50% probability level.

## supplementary crystallographic information

### Comment

2,3-Dihydropyrimido[4,5-*d*]pyrimidin-4(1*H*)-ones constitute a class of fused heterocycles which possess anti-cancer (McDermott *et al.*, 2006) and anti-bacterial activity (Gebauer *et al.*, 2003). 2-Substituted 2,3-dihydropyrimido[4,5-*d*]pyrimidin-4(1*H*)-one derivatives can be obtained from the cyclocondensation of 2,4-diaminopyrimidine-5-carbonitrile with cycloheptanone. Here, we report the crystal structure of the title compound (Fig. 1).

The molecular structure (Fig. 1) is built up with two fused six-membered ring and one seven-membered ring linked through a spiro C atom. The hydropyrimidine ring has a distorted bath conformation, similar to that found in Spiro{cyclopentane-1,2'(1'H)pyrido [2',3'-d]pyrimidin-4'(3'H)-one} (Shi *et al.*, 2010). The crystal packing is stabilized by intermolecular N–H···O hydrogen bonds between the two N–H groups and the ketone O atoms of the neighbouring molecules (Table 1).

### Experimental

A solution of 2,4-diaminopyrimidine-5-carbonitrile (2 mmol) and sodium methylate (2 mmol) was refluxed in cycloheptanone (3 ml) for 6 h. The reaction mixture was cooled to room temperature and then filtered to give the title compound. The product was recrystallizated from methanol to give light yellow crystalline powder.

Spectral data: IR (KBr): 3413, 3339, 3173, 2933, 1659, 1624, 1600, 1473, 1408 cm^-1^; ^1^H-NMR(DMSO,p.p.m.):1.57 (8H, s,CH), 1.79-1.90 (4H, m, CH), 6.60 (2H, s, NH_2_), 7.856 (1H, s, pyrimidine-NH), 7.865 (1H, s, pyrimidine-H), 8.168 (1H, s, NH-CO); ESI-MS m/z: [M+H]^+^ 248.1.

### Crystal data

**Table:**

C_12_H_17_N_5_O	*F*(000) = 528
*M**_r_* = 247.31	*D*_x_ = 1.380 Mg m^−^^3^
Monoclinic, *P*2_1_/*n*	Mo *K*α radiation, λ = 0.71073 Å
*a* = 10.798 (3) Å	Cell parameters from 4105 reflections
*b* = 10.365 (3) Å	θ = 2.0–30.0°
*c* = 11.341 (3) Å	µ = 0.09 mm^−^^1^
β = 110.287 (4)°	*T* = 153 K
*V* = 1190.5 (6) Å^3^	Block, colourless
*Z* = 4	0.39 × 0.35 × 0.26 mm

### Data collection

**Table:**

Rigaku AFC10/Saturn724+ diffractometer	3237 reflections with *I* > 2σ(*I*)
Radiation source: Rotating Anode	*R*_int_ = 0.036
Graphite monochromator	θ_max_ = 30.0°, θ_min_ = 2.2°
Detector resolution: 28.5714 pixels mm^-1^	*h* = −15→15
phi and ω scans	*k* = −14→10
9163 measured reflections	*l* = −9→15
3450 independent reflections	

### Refinement

**Table:**

Refinement on *F*^2^	Secondary atom site location: difference Fourier map
Least-squares matrix: full	Hydrogen site location: inferred from neighbouring sites
*R*[*F*^2^ > 2σ(*F*^2^)] = 0.054	H atoms treated by a mixture of independent and constrained refinement
*wR*(*F*^2^) = 0.129	*w* = 1/[σ^2^(*F*_o_^2^) + (0.0495*P*)^2^ + 0.960*P*] where *P* = (*F*_o_^2^ + 2*F*_c_^2^)/3
*S* = 1.00	(Δ/σ)_max_ < 0.001
3450 reflections	Δρ_max_ = 0.35 e Å^−^^3^
180 parameters	Δρ_min_ = −0.22 e Å^−^^3^
0 restraints	Extinction correction: *SHELXL97* (Sheldrick, 2008)
Primary atom site location: structure-invariant direct methods	Extinction coefficient: 0.0058 (19)

### Special details

**Table:**

Geometry. All e.s.d.'s (except the e.s.d. in the dihedral angle between two l.s. planes) are estimated using the full covariance matrix. The cell e.s.d.'s are taken into account individually in the estimation of e.s.d.'s in distances, angles and torsion angles; correlations between e.s.d.'s in cell parameters are only used when they are defined by crystal symmetry. An approximate (isotropic) treatment of cell e.s.d.'s is used for estimating e.s.d.'s involving l.s. planes.
Refinement. Refinement of *F*^2^ against ALL reflections. The weighted *R*-factor *wR* and goodness of fit *S* are based on *F*^2^, conventional *R*-factors *R* are based on *F*, with *F* set to zero for negative *F*^2^. The threshold expression of *F*^2^ > σ(*F*^2^) is used only for calculating *R*-factors(gt) *etc*. and is not relevant to the choice of reflections for refinement. *R*-factors based on *F*^2^ are statistically about twice as large as those based on *F*, and *R*- factors based on ALL data will be even larger.

### Fractional atomic coordinates and isotropic or equivalent isotropic displacement parameters (Å^2^)

**Table:**

	*x*	*y*	*z*	*U*_iso_*/*U*_eq_	
O1	0.50270 (10)	0.09029 (10)	0.36576 (9)	0.0190 (2)	
N1	0.59501 (11)	0.15243 (11)	0.56975 (10)	0.0168 (2)	
N2	0.76803 (11)	0.30528 (12)	0.63161 (10)	0.0180 (2)	
N3	0.83812 (11)	0.40875 (11)	0.48490 (10)	0.0175 (2)	
N4	0.71560 (11)	0.38221 (12)	0.26293 (10)	0.0190 (2)	
N5	0.90263 (12)	0.50418 (13)	0.33343 (12)	0.0217 (3)	
C1	0.55839 (13)	0.36082 (13)	0.65798 (12)	0.0190 (3)	
H1A	0.6087	0.4380	0.6996	0.023*	
H1B	0.5115	0.3832	0.5687	0.023*	
C2	0.45569 (14)	0.33082 (16)	0.71849 (13)	0.0235 (3)	
H2A	0.4361	0.2372	0.7106	0.028*	
H2B	0.3730	0.3774	0.6723	0.028*	
C3	0.50040 (16)	0.36863 (18)	0.85753 (14)	0.0302 (4)	
H3A	0.5270	0.4605	0.8652	0.036*	
H3B	0.4239	0.3608	0.8861	0.036*	
C4	0.61375 (15)	0.29004 (18)	0.94541 (13)	0.0281 (3)	
H4A	0.5803	0.2028	0.9536	0.034*	
H4B	0.6421	0.3307	1.0296	0.034*	
C5	0.73471 (14)	0.27534 (16)	0.90615 (12)	0.0235 (3)	
H5A	0.8085	0.2403	0.9779	0.028*	
H5B	0.7615	0.3617	0.8862	0.028*	
C6	0.71173 (14)	0.18714 (14)	0.79249 (12)	0.0198 (3)	
H6A	0.6495	0.1182	0.7958	0.024*	
H6B	0.7966	0.1452	0.8002	0.024*	
C7	0.65745 (12)	0.25199 (13)	0.66346 (11)	0.0150 (2)	
C8	0.57409 (12)	0.16677 (13)	0.44658 (12)	0.0153 (2)	
C9	0.64680 (12)	0.27115 (13)	0.41465 (12)	0.0158 (2)	
C10	0.75182 (12)	0.33111 (13)	0.51061 (12)	0.0154 (2)	
C11	0.81680 (12)	0.42899 (13)	0.36179 (12)	0.0167 (3)	
C12	0.63510 (13)	0.30267 (13)	0.29228 (12)	0.0177 (3)	
H12	0.5650	0.2648	0.2256	0.021*	
H2N	0.8326 (19)	0.3443 (19)	0.6896 (18)	0.026 (5)*	
H0A	0.9754 (19)	0.5290 (18)	0.3921 (18)	0.023 (4)*	
H0B	0.893 (2)	0.514 (2)	0.257 (2)	0.033 (5)*	
H1N	0.557 (2)	0.087 (2)	0.593 (2)	0.035 (5)*	

### Atomic displacement parameters (Å^2^)

**Table:**

	*U*^11^	*U*^22^	*U*^33^	*U*^12^	*U*^13^	*U*^23^
O1	0.0220 (5)	0.0204 (5)	0.0161 (4)	−0.0067 (4)	0.0087 (4)	−0.0042 (3)
N1	0.0218 (5)	0.0164 (5)	0.0142 (5)	−0.0044 (4)	0.0089 (4)	−0.0008 (4)
N2	0.0165 (5)	0.0254 (6)	0.0122 (5)	−0.0068 (4)	0.0050 (4)	−0.0011 (4)
N3	0.0180 (5)	0.0208 (6)	0.0151 (5)	−0.0047 (4)	0.0075 (4)	−0.0006 (4)
N4	0.0220 (5)	0.0207 (6)	0.0147 (5)	−0.0036 (4)	0.0066 (4)	0.0003 (4)
N5	0.0217 (6)	0.0278 (6)	0.0166 (5)	−0.0085 (5)	0.0077 (4)	0.0009 (5)
C1	0.0207 (6)	0.0194 (6)	0.0166 (6)	0.0011 (5)	0.0062 (5)	−0.0008 (5)
C2	0.0193 (6)	0.0331 (8)	0.0189 (6)	0.0024 (5)	0.0076 (5)	−0.0015 (5)
C3	0.0295 (7)	0.0423 (10)	0.0217 (7)	0.0034 (7)	0.0127 (6)	−0.0065 (6)
C4	0.0288 (7)	0.0405 (9)	0.0162 (6)	−0.0032 (6)	0.0091 (5)	−0.0045 (6)
C5	0.0209 (6)	0.0346 (8)	0.0129 (6)	−0.0037 (5)	0.0033 (5)	0.0000 (5)
C6	0.0217 (6)	0.0234 (7)	0.0151 (6)	0.0022 (5)	0.0074 (5)	0.0044 (5)
C7	0.0157 (5)	0.0180 (6)	0.0121 (5)	−0.0026 (4)	0.0057 (4)	−0.0007 (4)
C8	0.0158 (5)	0.0166 (6)	0.0151 (5)	−0.0011 (4)	0.0073 (4)	−0.0017 (4)
C9	0.0173 (5)	0.0171 (6)	0.0137 (5)	−0.0029 (4)	0.0064 (4)	−0.0009 (4)
C10	0.0162 (5)	0.0171 (6)	0.0140 (5)	−0.0006 (4)	0.0065 (4)	−0.0001 (4)
C11	0.0181 (6)	0.0174 (6)	0.0163 (6)	−0.0011 (4)	0.0080 (5)	0.0004 (4)
C12	0.0190 (6)	0.0196 (6)	0.0142 (5)	−0.0026 (5)	0.0054 (4)	−0.0012 (4)

### Geometric parameters (Å, º)

**Table:**

O1—C8	1.2541 (16)	C2—H2A	0.9900
N1—C8	1.3437 (17)	C2—H2B	0.9900
N1—C7	1.4670 (16)	C3—C4	1.520 (2)
N1—H1N	0.88 (2)	C3—H3A	0.9900
N2—C10	1.3488 (17)	C3—H3B	0.9900
N2—C7	1.4698 (16)	C4—C5	1.527 (2)
N2—H2N	0.87 (2)	C4—H4A	0.9900
N3—C10	1.3376 (16)	C4—H4B	0.9900
N3—C11	1.3505 (17)	C5—C6	1.529 (2)
N4—C12	1.3215 (17)	C5—H5A	0.9900
N4—C11	1.3552 (17)	C5—H5B	0.9900
N5—C11	1.3328 (17)	C6—C7	1.5304 (18)
N5—H0A	0.87 (2)	C6—H6A	0.9900
N5—H0B	0.84 (2)	C6—H6B	0.9900
C1—C2	1.525 (2)	C8—C9	1.4543 (18)
C1—C7	1.5408 (19)	C9—C12	1.3883 (18)
C1—H1A	0.9900	C9—C10	1.4144 (17)
C1—H1B	0.9900	C12—H12	0.9500
C2—C3	1.531 (2)		
			
C8—N1—C7	123.01 (11)	H4A—C4—H4B	107.4
C8—N1—H1N	118.1 (14)	C4—C5—C6	113.60 (12)
C7—N1—H1N	118.0 (14)	C4—C5—H5A	108.8
C10—N2—C7	119.65 (10)	C6—C5—H5A	108.8
C10—N2—H2N	117.5 (13)	C4—C5—H5B	108.8
C7—N2—H2N	119.5 (13)	C6—C5—H5B	108.8
C10—N3—C11	115.90 (11)	H5A—C5—H5B	107.7
C12—N4—C11	115.27 (11)	C5—C6—C7	116.11 (12)
C11—N5—H0A	120.4 (12)	C5—C6—H6A	108.3
C11—N5—H0B	118.1 (14)	C7—C6—H6A	108.3
H0A—N5—H0B	120.3 (19)	C5—C6—H6B	108.3
C2—C1—C7	115.82 (12)	C7—C6—H6B	108.3
C2—C1—H1A	108.3	H6A—C6—H6B	107.4
C7—C1—H1A	108.3	N1—C7—N2	107.14 (10)
C2—C1—H1B	108.3	N1—C7—C6	108.14 (11)
C7—C1—H1B	108.3	N2—C7—C6	109.00 (10)
H1A—C1—H1B	107.4	N1—C7—C1	110.33 (10)
C1—C2—C3	113.10 (12)	N2—C7—C1	109.07 (11)
C1—C2—H2A	109.0	C6—C7—C1	112.98 (11)
C3—C2—H2A	109.0	O1—C8—N1	121.95 (12)
C1—C2—H2B	109.0	O1—C8—C9	122.43 (12)
C3—C2—H2B	109.0	N1—C8—C9	115.49 (11)
H2A—C2—H2B	107.8	C12—C9—C10	115.88 (12)
C4—C3—C2	115.65 (13)	C12—C9—C8	123.65 (11)
C4—C3—H3A	108.4	C10—C9—C8	119.55 (11)
C2—C3—H3A	108.4	N3—C10—N2	119.12 (11)
C4—C3—H3B	108.4	N3—C10—C9	122.00 (12)
C2—C3—H3B	108.4	N2—C10—C9	118.83 (12)
H3A—C3—H3B	107.4	N5—C11—N3	117.18 (12)
C3—C4—C5	115.99 (13)	N5—C11—N4	115.99 (12)
C3—C4—H4A	108.3	N3—C11—N4	126.82 (12)
C5—C4—H4A	108.3	N4—C12—C9	123.97 (12)
C3—C4—H4B	108.3	N4—C12—H12	118.0
C5—C4—H4B	108.3	C9—C12—H12	118.0
			
C7—C1—C2—C3	90.57 (16)	O1—C8—C9—C12	5.9 (2)
C1—C2—C3—C4	−67.94 (19)	N1—C8—C9—C12	−178.21 (12)
C2—C3—C4—C5	50.1 (2)	O1—C8—C9—C10	−162.74 (13)
C3—C4—C5—C6	−71.13 (19)	N1—C8—C9—C10	13.19 (18)
C4—C5—C6—C7	88.34 (16)	C11—N3—C10—N2	179.16 (12)
C8—N1—C7—N2	−41.57 (16)	C11—N3—C10—C9	1.64 (19)
C8—N1—C7—C6	−158.93 (12)	C7—N2—C10—N3	162.87 (12)
C8—N1—C7—C1	77.05 (15)	C7—N2—C10—C9	−19.52 (19)
C10—N2—C7—N1	42.90 (16)	C12—C9—C10—N3	−2.94 (19)
C10—N2—C7—C6	159.69 (12)	C8—C9—C10—N3	166.53 (12)
C10—N2—C7—C1	−76.53 (15)	C12—C9—C10—N2	179.53 (12)
C5—C6—C7—N1	−158.50 (11)	C8—C9—C10—N2	−11.01 (19)
C5—C6—C7—N2	85.34 (14)	C10—N3—C11—N5	−178.86 (12)
C5—C6—C7—C1	−36.09 (16)	C10—N3—C11—N4	2.2 (2)
C2—C1—C7—N1	76.85 (14)	C12—N4—C11—N5	176.75 (13)
C2—C1—C7—N2	−165.72 (11)	C12—N4—C11—N3	−4.3 (2)
C2—C1—C7—C6	−44.33 (15)	C11—N4—C12—C9	2.6 (2)
C7—N1—C8—O1	−168.59 (12)	C10—C9—C12—N4	0.7 (2)
C7—N1—C8—C9	15.46 (18)	C8—C9—C12—N4	−168.33 (13)

### Hydrogen-bond geometry (Å, º)

**Table:**

*D*—H···*A*	*D*—H	H···*A*	*D*···*A*	*D*—H···*A*
N1—H1*N*···O1^i^	0.88 (2)	2.05 (2)	2.9176 (16)	167 (2)
N2—H2*N*···O1^ii^	0.87 (2)	2.30 (2)	3.1587 (16)	168.3 (17)
N5—H0*B*···O1^iii^	0.84 (2)	2.22 (2)	2.9234 (17)	141.4 (19)
N5—H0*A*···N3^iv^	0.87 (2)	2.11 (2)	2.9826 (18)	172.5 (17)

Symmetry codes: (i) −*x*+1, −*y*, −*z*+1; (ii) *x*+1/2, −*y*+1/2, *z*+1/2; (iii) −*x*+3/2, *y*+1/2, −*z*+1/2; (iv) −*x*+2, −*y*+1, −*z*+1.
